# Immune-Related Gene SERPINE1 Is a Novel Biomarker for Diffuse Lower-Grade Gliomas *via* Large-Scale Analysis

**DOI:** 10.3389/fonc.2021.646060

**Published:** 2021-05-20

**Authors:** Xiaoming Huang, Fenglin Zhang, Dong He, Xiaoshuai Ji, Jiajia Gao, Wenqing Liu, Yunda Wang, Qian Liu, Tao Xin

**Affiliations:** ^1^ Department of Neurosurgery, Shandong Provincial Qianfoshan Hospital, Cheeloo College of Medicine, Shandong University, Jinan, China; ^2^ Department of Neurosurgery, Shandong Provincial Hospital, Cheeloo College of Medicine, Shandong University, Jinan, China; ^3^ Department of Neurosurgery, Shandong Provincial Qianfoshan Hospital, Shandong First Medical University & Shandong Academy of Medical Sciences, Jinan, China; ^4^ Department of Histology and Embryology, School of Basic Medical Sciences, Cheeloo College of Medicine, Shandong University, Jinan, China; ^5^ Department of Neurosurgery, Jiangxi Provincial People’s Hospital Affiliated to Nanchang University, Nanchang, Jiangxi, China; ^6^ Shandong Medicine and Health Key Laboratory of Neurosurgery, The First Affiliated Hospital of Shandong First Medical University & Shandong Provincial Qianfoshan Hospital, Jinan, China

**Keywords:** SERPINE1, LGG, TME, biomarker, immune checkpoint, prognosis

## Abstract

**Background:**

Glioma is one of the highly fatal primary tumors in the central nervous system. As a major component of tumor microenvironment (TME), immune cell has been proved to play a critical role in the progression and prognosis of the diffuse lower-grade gliomas (LGGs). This study aims to screen the key immune-related factors of LGGs by investigating the TCGA database.

**Methods:**

The RNA-sequencing data of 508 LGG patients were downloaded in the TCGA database. ESTIMATE algorithm was utilized to calculate the stromal, immune, and ESTIMATE scores, based on which, the differentially expressed genes (DEGs) were analyzed by using “limma” package. Cox regression analysis and the cytoHubba plugin of Cytoscape software were subsequently applied to screen the survival-related genes and hub genes, the intersection of which led to the identification of SERPINE1 that played key roles in the LGGs. The expression patterns, clinical features, and regulatory mechanisms of SERPINE1 in the LGGs were further analyzed by data mining of the TCGA database. What’s more, the above analyses of SERPINE1 were further validated in the LGG cohort from the CGGA database.

**Result:**

We found that stromal and immune cell infiltrations were strongly related to the prognosis and malignancy of the LGGs. A total of 54 survival-related genes and 46 hub genes were screened out in the DEGs, within which SERPINE1 was identified to be significantly overexpressed in the LGG samples compared with the normal tissues. Moreover, the upregulation of SERPINE1 was more pronounced in the gliomas of WHO grade III and IDH wild type, and its expression was correlated with poor prognosis in the LGG patients. The independent prognostic value of SERPINE1 in the LGG patients was also confirmed by Cox regression analysis. In terms of the functions of SERPINE1, the results of enrichment analysis indicated that SERPINE1 was mainly enriched in the immune‐related biological processes and signaling pathways. Furthermore, it was closely associated with infiltrations of immune cells in the LGG microenvironment and acted synergistically with PD1, PD-L1, PD-L2.

**Conclusion:**

These findings proved that SERPINE1 could serve as a prognostic biomarker and potential immunotherapy target of LGGs.

## Introduction

Glioma is the most common type of primary brain tumors in the central nervous system (CNS), originated from the transformed progenitor cells or neural stem cells (1). According to the WHO Classification of CNS tumors revised in 2016, gliomas are divided into four grades from WHO grade I to WHO grade IV and comprise two major subtypes: diffuse gliomas and gliomas and non-diffuse gliomas (2, 3). Among them, diffuse low-grade (WHO grade II) and intermediate-grade (WHO grade III) gliomas are collectively referred to as lower-grade gliomas (LGGs) (4). Compared with glioblastoma (WHO grade IV), LGGs are relatively benign and have a favorable prognosis. However, LGGs commonly exhibit diffuse and infiltrative nature, which makes it extremely difficult to be completely resected. As a matter of fact, most LGGs eventually progress to the secondary tumors with higher grades (4, 5). Despite ongoing advances in surgical operation and postoperative adjuvant chemoradiotherapy, the prognosis of gliomas has not been dramatically improved over the past decades (6, 7). Therefore, more effective therapeutic strategies for LGGs need to be further explored.

The tumor microenvironment (TME), which consists of large and diverse amounts of immune cells, stromal cells and other non-tumor components, plays pivotal roles in tumor initiation and progression (8, 9). For instance, tumor-associated macrophages (TAM) affect the development of tumors mainly through proliferation, local infiltration, angiogenesis and immunosuppression (10, 11). Other immune cells, including effector T cells, regulatory T cells, B cells, natural killer (NK) cells, dendritic cells (DCs), and N1-polarized neutrophils, have also been reported to serve various functions in the TME (12). In recent years, tumor immunotherapies that target tumor or immune cells have evolved to the most promising therapeutic approaches to treat cancers (13, 14). Immune checkpoint inhibitors, such as programmed cell death receptor 1 (PD-1) inhibitor, programmed death-ligand 1 (PD-L1) inhibitor, have made tremendous progress in the clinical treatment of melanoma, non–small cell lung cancer (NSCLC), and urothelial carcinoma (UC) (15–17). However, immune suppression and evasion that also exist in the TME remain the formidable challenges to effective immunotherapies in some tumor patients (18). For example, under induction of CSF-1, CCL2, IL-4, IL-6, IL-10, tumor-associated macrophages (TAMs) in the glioma microenvironment differentiate into M2-type macrophages (19, 20), which contribute to establishing an immunosuppressive microenvironment due to lack of costimulatory factors such as CD40, CD80, and CD86 (21, 22). Hence, further investigation of the immune status of the TME remains particularly needed.

Serpin family E member 1 (SERPINE1), encoding plasminogen activator inhibitor 1 (PAI-1), serves as the primary inhibitor of uridylyl phosphate adenosine (uPA) and tissue plasminogen activator (tPA) (23). Previous researches have predominantly focused on its function in thrombosis (24). With the recent development of high-throughput sequencing technology, the abnormal expression of SERPINE1 has been detected in various tumors and its role in tumors has attracted great attention. SERPINE1 has been reported to induce tumor migration, invasion, angiogenesis and thereby promote the progression and metastasis of tumors (24, 25). For example, SERPINE1 was reported to be elevated in the gastric adenocarcinoma tissues and its upregulation enhanced the invasive and proliferative capacities of tumor cells by regulating epithelial-mesenchymal transition (EMT) (26). Moreover, SERPINE1 was identified as a regulator of glioblastoma cell dispersal and downregulation of SERPINE1 limited the proliferation and invasion of glioma cells (27). However, the specific molecular mechanisms underlying these phenotypes caused by SERPINE1 in gliomas still remain obscure.

In this study, the LGG cohort data from the TCGA database was mined to screen the prognostic immune‐related genes for the LGGs. SERPINE1 was finally determined as our research objective. The association between the expressions and clinical features of SERPINE1were analyzed *via* using the LGG RNA-seq data from the TCGA and CGGA database. To better elucidate the biological mechanisms of SERPINE1, we carried out the gene co-expression analysis, GSEA, immune-cell infiltration correlation analysis, and immune checkpoints correlation analysis in the LGG cohort. Finally, we proved that SERPINE1 served as an oncogene in the LGGs and might be a novel potential target for glioma immunotherapy.

## Materials and Methods

### Data Collection and Processing

The TCGA RNA-seq data and corresponding phenotype data of LGG samples were downloaded from UCSC Xena website (http://xena.ucsc.edu/). Samples with incomplete information and duplicates were removed. Stromal scores and immune scores were calculated by the ESTIMATE algorithm for each sample (28). RNA-seq data and corresponding clinical information used for further validation were downloaded from the CGGA database (29, 30). Batch effects were removed using the “sva” Bioconductor package (31). We extracted the following clinical characteristics for this study: gender, age, survival status, survival time, tumor grade, IDH status. The glioma tissue chip was purchased from the Shanghai Outdo Biotech Co. Ltd (Shanghai, China), which contained 30 LGG cases (Lot No.: XT16-017).

### Screening Differentially Expressed Genes (DEGs)

All LGG patients were divided into high/low groups according to the immune scores and stromal scores. We screened the DEGs between the high and low score groups using the “limma” R package, with the thresholds of p-value<0.05 and log2|fold change|> 1 (32). The intersections of these DEG sets were showed by Venn diagram (http://bioinformatics.psb.ugent.be/webtools/Venn/).

### Functional Enrichment Analysis

Kyoto Encyclopedia of Genes and Genomes (KEGG) analysis and gene ontology (GO) analysis were performed using the Bioconductor package “clusterProfiler” to identify the possible pathways and functions of the DEGs (33). GO analysis included three categories: cellular component (CC), biological process (BP), and molecular function (MF). Metascape database was utilized to conduct functional enrichment analysis for the top 500 SERPINE1 positively correlated genes (34). The terms with p-value<0.05 were considered statistically significant.

### PPI Network, GSEA, GSVA, and ROC

The protein-protein interaction (PPI) network was constructed using the STRING online tool (35) and visualized by Cytoscape software (V3.7.1) (36). Gene set enrichment analysis (GSEA) was performed using GSEA software (V 4.1.0). The false discovery rate (FDR)<0.05 was considered statistically significant. Gene set variation analysis (GSVA) was performed *via* R software (37). The receiver operating characteristic (ROC) curves were constructed using “survivalROC” package (38).

### Tumor-Infiltrating Immune Cells

To calculate the abundance of 22 immune cell types in each LGG sample, we submitted the gene expression data to the CIBERSORT website (https://cibersort.stanford.edu/) and performed the CIBERSORT deconvolution algorithm (39). The results with p-value<0.05 were considered statistically significant.

### Immunohistochemistry (IHC)

PAI-1 and cell markers were detected by immunohistochemistry (ICH) that was conducted with the standard protocol. Rabbit anti-PAI1 antibody was purchased from ZEN-BIOSCIENCE (Chengdu, Sichuan, China), and other primary antibodies were purchased from Affinity Biosciences LTD. Secondary antibody (HRP conjugated Goat Anti-Rabbit IgG) was purchased from Servicebio Technology Co. Ltd (Wu Han, China). Primary antibodies and secondary antibody were respectively diluted at a ratio of 1:50 and 1:200. The staining intensity of each staining area was categorized into four-level: negative staining (scored 0), weak staining (scored 1), moderate staining (scored 2), strong staining (scored 3). And the area of each staining intensity was measured respectively. We quantified the results of tissue microarray immunohistochemistry staining using histochemistry score (H-score). H-score = (percentage of weak staining area ×1) + (percentage of moderate staining area ×2) + (percentage of strong staining area ×3).

### Statistical Analysis and Plot Generation

The R software (Version 4.0.3), GraphPad Prism 8 software (Version 8.0.2), and Adobe Illustrator software (Version 24.0.2) were used to perform statistical analysis and generate figures. Kaplan-Meier survival analysis was performed using the “survival” (https://CRAN.R-project.org/package=survival) and “survminer” (Version: 0.4.8) R packages. Wilcoxon rank-sum test was used to compare the median values between the variables. The Cox regression model was used for univariate and multivariate analyses. We calculated the correlations between the different variables *via* the Spearman correlation test. In all statistical tests, p-value<0.05 was considered statistically significant. The plots were generated by R packages: “ggplot2” (Version: 3.3.2), “ggpubr” (Version: 0.4.0), “pheatmap” (Version: 1.0.12), “VennDiagram” (Version: 1.6.20), “enrichplot” (40), “survivalROC” (Version: 1.0.3), “vioplot” (Version: 0.3.5), “corrplot” (Version: 0.84).

## Results

### Relationship Between The Immune, Stromal, ESTIMATE Scores and the Clinical Characteristics of the LGG Patients

**Figure 1:**
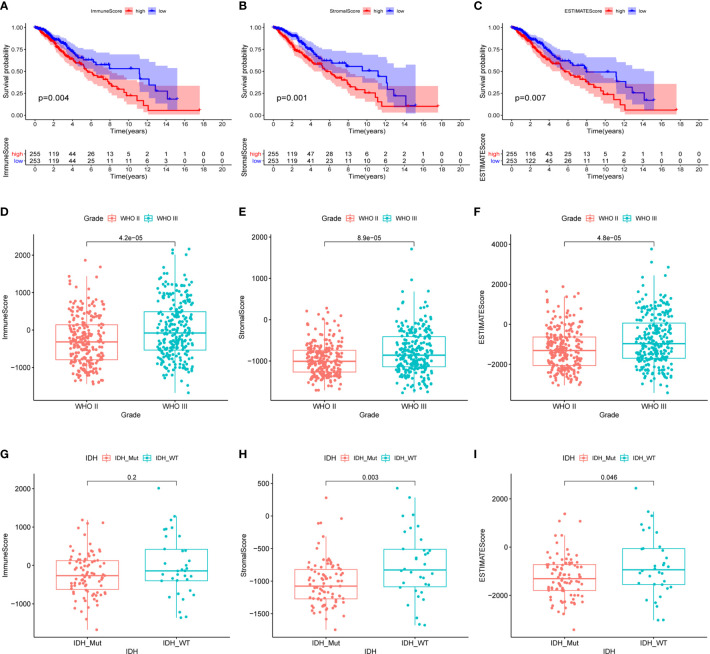
Relationship between the immune, stromal, ESTIMATE scores and the clinical characteristics of the LGG patients. **(A)** Kaplan-Meier survival analysis of LGG patients in high and low immune-score groups. **(B)** Kaplan-Meier survival analysis of LGG patients in high and low stromal-score groups. **(C)** Kaplan-Meier survival analysis of LGG patients in high and low ESTIMATE-score groups. **(D–F)** The distribution of immune score, stromal score, ESTIMATE score in tumor grades. **(G–I)** The distribution of immune score, stromal score, ESTIMATE score in IDH status. P-value < 0.05 was considered statistically significant.

The gene expression profiling data and clinical information of 533 LGG samples were downloaded from the TCGA database (https://www.cancer.gov/tcga). Three samples, including TCGA-TQ-A7RS-01A, TCGA-CS-5390-01A, and TCGA-R8-A6YH-01A, were excluded from our study cohort for lack of complete clinical information. Since 18 patients in the cohort corresponded to multiple sample information, we thus merged these repeated gene expression profiles after taking the average. Ultimately, a total of 508 LGG patients were enrolled in our study, and their clinical informations were presented in **Table 1**. Based on the ESTIMATE algorithm, the immune scores varied from -1676.002 to 2477.026, the stromal scores ranged from -1769.170 to 1710.690, and the ESTIMATE scores (ESTIMATE score is the sum of the immune score and stromal score of each sample, which reflects the purity of the tumor. The higher the ESTIMATE score, the lower the purity of the tumor.) were distributed between -3422.599 and 3762.907 (**Supplementary Table 1**). We subsequently sorted the 508 LGG cases into high-score and low-score groups according to the median value of these scores. Kaplan-Meier analysis indicated that the cases with low immune (p=0.004), stromal (p=0.001), and ESTIMATE (p=0.007) scores exhibited longer overall survival than those with high scores (**Figures 1A–C**). We further analyzed the associations between these scores and the clinical characteristics of the LGG patients. The results showed that the WHO grade III and IDH wild-type LGG patients exhibited higher immune, stromal, and ESTIMATE scores, although the immune score was not statistically significant between the IDH groups (**Figures 1D–I**). No significant differences also appeared in age and gender subgroups (**Supplementary Figures 1A-F**).

**Table 1:** Demographic and clinicopathologic characteristics of the LGG patients enrolled in this study.

		TCGA dataset	CGGA dataset
Total		508	575
Gender			
	male	282	334
	female	226	241
Event			
	alive	382	245
	dead	126	330
Age			
	age ≤ 41	265	323
	age >41	243	252
Grade			
	WHO II	247	271
	WHO III	261	304
Radiotherapy			
	yes	286	430
	no	176	128
	unknown	46	17
Chemotherapy (TMZ)			
	yes	–	359
	no	–	200
	unknown	–	16
Corticosteroids			
	non-treatment	211	–
	treatment	149	–
	unknown	148	–
IDH status			
	wild type	34	130
	mutation	91	411
	unknown	383	34
1p19q status			
	non-codeletion	–	366
	codeletion	–	175
	unknown	–	34
MGMTp status			
	methylated	–	279
	un-methylated	–	194
	unknown	–	102
KPS			
	KPS≥90	196	–
	70≤KPS<90	81	–
	KPS<70	22	–
	unknown	209	

KPS, Karnofsky performance score; TMZ, temozolomide; MGMTp, MGMT promoter.

### Identification of the Differentially Expressed Genes (DEGs) Based on the Immune and Stromal Scores of the LGGs

**Figure 2:**
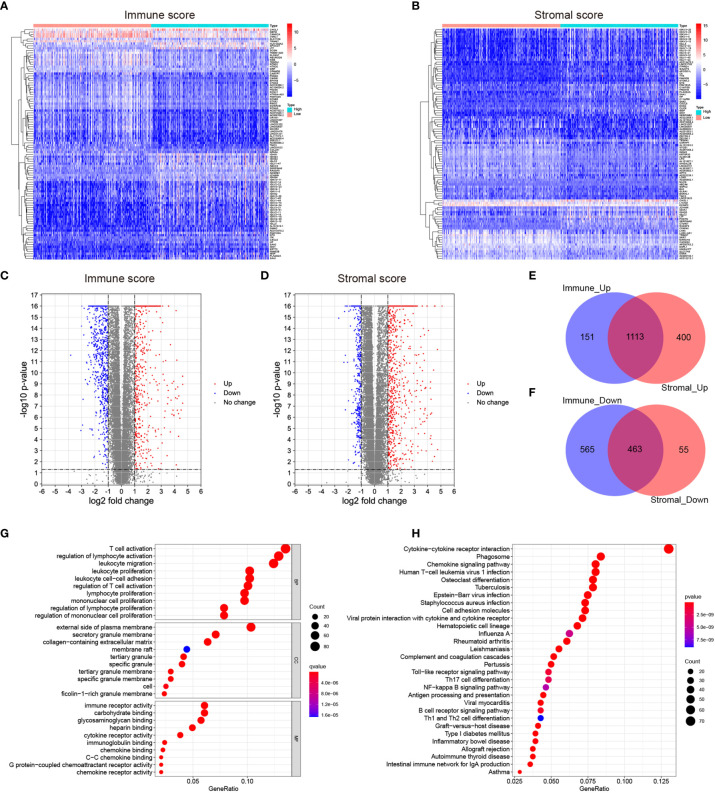
Identification of the differentially expressed genes (DEGs) based on the immune and stromal scores of the LGGs. **(A, B)** Heatmaps showing the distribution patterns of differentially expressed genes based on immune and stromal scores. The darker the red color, the higher the gene expression was. The deeper the blue color, the lower the gene expression was. **(C, D)** Volcano plots of significantly differentially expressed genes based on immune and stromal scores (|lg FC|>1, p < 0.05). Blue dots represented significantly down-regulated genes, and red dots represented significantly up-regulated genes. **(E, F)** Venn plots showing the overlapping DEGs between immune and stromal groups. **(G)** Bubble plot of GO enrichment analysis of DEGs. **(H)** Bubble plot of KEGG enrichment analysis of DEGs. Node size represented the number of DEGs contained in the corresponding GO/KEGG term, and the node color denoted the p-value. P-value<0.05 was considered statistically significant.

To identify the DEGs, the LGG patients were classified into the high-score and low-score groups based on the immune and stromal scores above. The DEGs were subsequently screened by comparing the gene expression profiles of the high-score and low-score groups, with a threshold of the absolute value of fold change > 2 (FDR<0.05). A total of 1264 up-regulated genes and 1028 down-regulated genes were selected in the high immune score group (**Figure 2C** and **Supplementary Table 2**). 1513 up-regulated genes and 518 down-regulated genes were chosen in the high stromal score group (**Figure 2D** and **Supplementary Table 2**). The “pheatmap” package was then employed to plot the heatmap, which exhibited the expression distribution of the DEGs between the high-score and low-score groups (**Figures 2A, B**). The intersected genes that were upregulated or downregulated in both immune and stromal groups were selected for further investigation (**Figures 2E, F**). Ultimately, a total of 1113 up-regulated genes and 463 down-regulated genes were included for the subsequent research.

To validate the potential functions of these 1576 DEGs, we performed Gene Ontology (GO) and Kyoto Encyclopedia of Genes and Genome (KEGG) pathway analyses. As presented in the bubble plot, the top GO terms enriched by these DEGs included T cell activation (BP), regulation of lymphocyte activation (BP), external side of plasma membrane (CC), and immune receptor activity (MF) (**Figure 2G**). On the other hand, the KEGG enrichment analysis indicated that the 1576 DEGs were predominantly enriched in the pathways of cytokine-cytokine receptor interaction, phagosome, and chemokine signaling pathway (**Figure 2H**). From the enrichment analysis results above, these DEGs were mainly involved in a varied range of biological processes and pathways associated with immune responses. Considering that these DEGs were obtained based on the immune and stromal cell scores, we thus identified the 1576 DEGs as immune-related genes (IRGs).

### Screening of Target Genes

**Figure 3:**
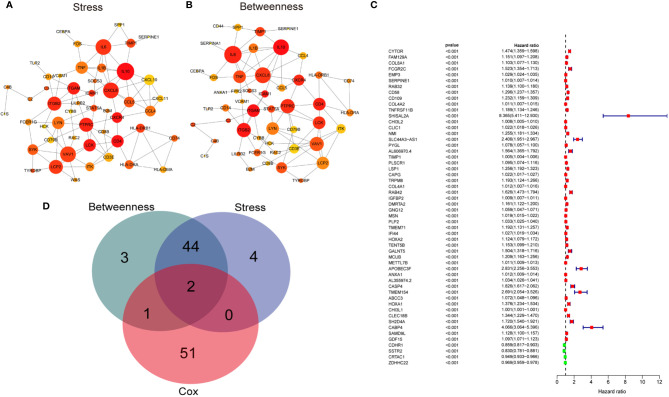
Screening target genes. **(A)** The network of top 50 hub genes screened by CytoHubba Stress algorithm. **(B)** The network of top 50 hub genes screened by CytoHubba Betweenness algorithm. Node size indicated its connectivity (the larger the node, the more proteins interacted). Node color indicated gene ranking (top-ranked genes colored by relatively darker color). **(C)** Forest plot showing hazard ratios with p‐values and 95% CI measured by univariate Cox regression analysis. The dashed line marked hazard ratio=1. Red boxes represented unfavorable prognosis genes, and blue boxes remarked favorable prognosis genes. CI, confidence interval. **(D)** Venn plots showing the intersected genes between hub genes and survival-related genes.

Initially, to gain the hub genes within the 1576 DEGs, the protein-protein interaction (PPI) network was constructed using the STRING database and visualized by Cytoscape software (v3.7.2) (**Supplementary Figure 2**). 46 hub genes in the network were identified by two algorithms (Stress and Betweenness) in the cytoHubba plugin of Cytoscape software (**Figures 3A, B**). Secondly, we conducted a univariate Cox regression analysis for the TCGA cohort to identify the genes correlated with the overall survival of the LGG patients. Of the 1576 DEGs that were analyzed, 54 genes were significantly associated with the prognosis of the LGG patients (p<0.001) (**Figure 3C**). Finally, the intersection of the 54 prognostic genes and the 46 hub genes led to the identification of SERPINE1 and TIMP1 (**Figure 3D**). Based on the online GEPIA2 database (http://gepia2.cancer-pku.cn/#index), the expression of TIMP1 was not significantly different between the LGG and normal brain tissues (**Supplementary Figure 3A**), which implied a minor role of TIMP1 as a biomarker for the LGGs. Therefore, SERPINE1 was selected as the target gene for the later study.

### The Expressions of SERPINE1 Increased With the Grades of Gliomas and Was Upregulated in the IDH Wild-Type LGGs

The analysis based on the GEPIA2 database indicated that SERPINE1 was significantly upregulated in the LGG samples compared with the normal brain tissues (**Supplementary Figure 3B**). We subsequently examined the expression patterns of SERPINE1 in the LGGs using the RNA-seq data from the TCGA database, which was further validated by the RNA-seq data in the CGGA database. The CGGA RNA-seq datasets that included 693 and 325 glioma samples were collected and merged after the batch effects by the “sva” package were removed (**Supplementary Figure 3C**). We ultimately selected the 575 LGG samples with the complete follow-up information from the merged dataset for our research (**Table 1**).

**Figure 4:**
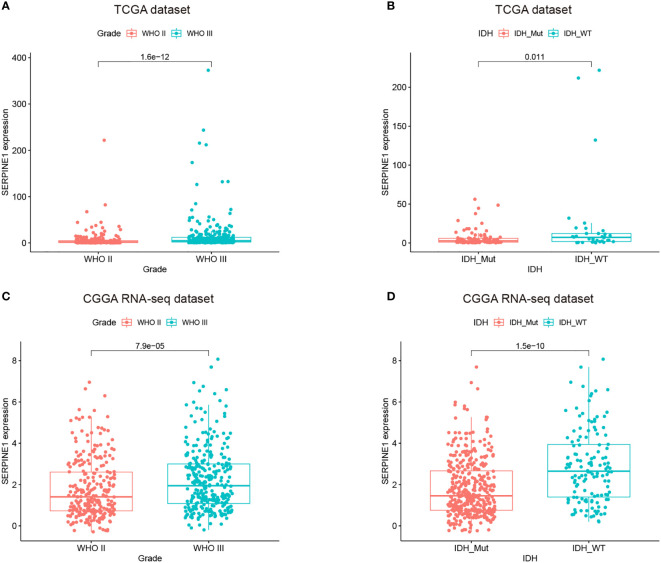
The expressions of SERPINE1 increased with the grades of gliomas and was upregulated in the IDH wild-type LGGs. **(A, C)** SERPINE1 was highly expressed in WHO grade III glioma. **(B, D)** SERPINE1 was significantly elevated in IDH wild-type glioma.

We found that the expression of SERPINE1 was comparatively higher in higher-grade tumors and patients of more advanced age. In the TCGA and CGGA cohorts, WHO grade III gliomas showed higher levels of SERPINE1 mRNA than WHO grade II gliomas (p<0.001, respectively) (**Figures 4A, C**). The older LGG patients tended to express higher levels of SERPINE1 mRNA (**Supplementary Figure 3D**) but failed to be validated in the CGGA cohort (**Supplementary Figure 3F**). It is well known that IDH status influences the prognosis of gliomas, among which IDH wild-type gliomas often associated with a worse survival rate (41). We thus determined the expression patterns of SERPINE1 based on IDH status. The results showed that the expression of SERPINE1 was significantly upregulated in the IDH wild-type gliomas in comparison to the IDH-mutant gliomas (**Figures 4B, D**). No significant difference in SERPINE1 mRNA levels was indicated by gender (**Supplementary Figures 3E, G**). In a word, the above results suggested that the expression of SERPINE1 was positively correlated with the malignancy of gliomas.

### SERPINE1 High Expression Predicted an Unfavorable Prognosis in the LGG Patients

**Figure 5:**
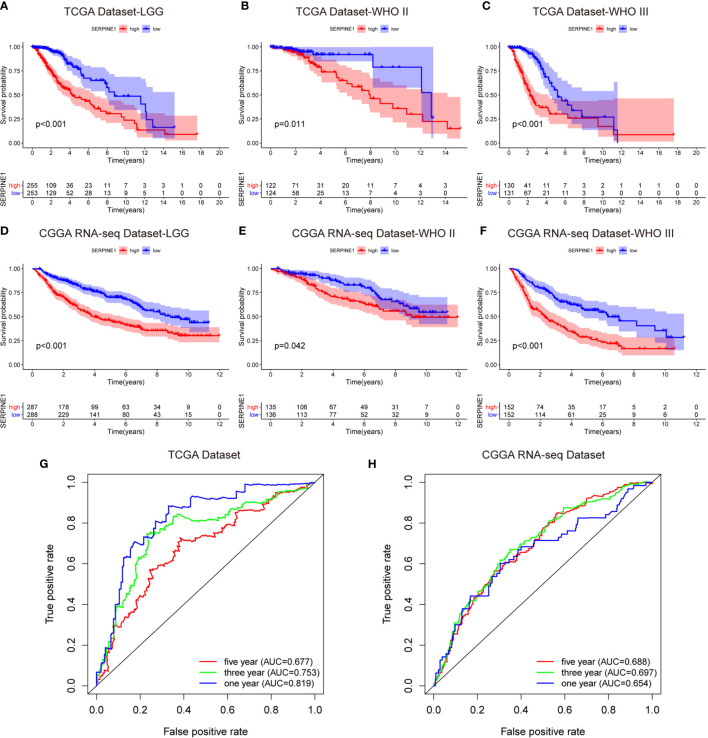
SERPINE1 high expression predicted an unfavorable prognosis in the LGG patients. **(A, D)** Kaplan-Meier overall survival analysis of SERPINE1 expression in all LGG patients. **(B, E)** Kaplan-Meier overall survival analysis of SERPINE1 expression in patients with grade II glioma. **(C, F)** Kaplan-Meier overall survival analysis of SERPINE1 expression in patients with grade III glioma. **(G, H)** ROC curve analysis of SERPINE1 in LGG patients. P-value < 0.05 was considered statistically significant.

To investigate the prognostic value of SERPINE1 for LGG patients, we collected the clinical and gene expression profile data from the TCGA and CGGA databases. The baseline of the patient characteristics was presented in **Table 1**. Firstly, the patients were divided into high and low expression groups based on the median value of SERPINE1 mRNA. The subsequent Kaplan-Meier analysis indicated that the high SERPINE1 expression group had shorter overall survival in all the LGG patients (P<0.001) (**Figure 5A**). Similar results were achieved in the WHO grade II patients (P=0.011) and WHO grade III patients (P<0.001) from the TCGA cohort (**Figures 5B, C**). In line with the results from the TCGA dataset, the patients with higher SERPINE1 expression also exhibited shorter overall survival in the CGGA dataset (P<0.001 for all LGGs, P=0.042 for WHO grade II gliomas, and P<0.001 for WHO grade III gliomas) (**Figures 5D–F**). Additionally, the univariate and multivariate Cox analysis of the TCGA and CGGA cohorts indicated that age, tumor grade, corticosteroids treatment, IDH status, 1p19q status, as well as SERPINE1 expression could serve as independent prognostic factors in patients with LGG (**Table 2**). Moreover, we performed receiver operating characteristic (ROC) curve analysis to assess the predictive ability (1-, 3-, 5-year overall survival) of SERPINE1 in LGG. The areas under the ROC curve (AUC) for 1-year survival were 0.819 in the TCGA cohort, 0.654 in the CGGA cohort; 3-year survival were 0.753 in the TCGA cohort, 0.697 in the CGGA cohort; 5-year survival were 0.677 in the TCGA cohort, 0.688 in the CGGA cohort (**Figures 5G, H**). All in all, the above results suggested that SERPINE1 could be an important prognostic biomarker for LGG patients.

**Table 2:** Univariate analysis and multivariate analysis of overall survival in the LGG cohort.

Datasets	Characteristic	Univariate			Multivariate		
		HR	95%CI	P.value	HR	95%CI	P.value
**TCGA**	Age	3.311	2.245-4.883	<0.001	3.110	2.090-4.627	0.009
	Gender	0.901	0.632-1.284	0.565	–	–	–
	Grade	3.434	2.323-5.076	<0.001	1.265	0.199-8.030	0.803
	Radiotherapy	2.001	1.289-3.131	0.002	14.639	0.821-261.099	0.068
	Corticosteroids	1.622	1.046-2.516	0.031	80.121	4.702-136.525	0.002
	IDH status	0.181	0.067-0.484	<0.001	0.067	0.008-0.516	0.009
	SERPINE1	1.010	1.006-1.014	<0.001	1.009	1.002-1.016	0.029
**CGGA**	Age	1.189	0.943-1.500	0.143	–	–	–
	Gender	1.112	0.881-1.404	0.372	–	–	–
	Grade	2.878	2.231-3.714	<0.001	3.177	2.418-4.175	<0.001
	Radiotherapy	1.011	0.756-1.351	0.943	–	–	–
	Chemotherapy	1.275	0.990-1.643	0.060	–	–	–
	IDH status	0.435	0.338-0.560	<0.001	0.694	0.525-0.917	0.001
	1p19q status	0.275	0.199-0.378	<0.001	0.319	0.226-0.449	<0.001
	MGMT status	0.795	0.618-1.024	0.075	–	–	–
	SERPINE1	1.008	1.006-1.011	<0.001	1.004	1.001-1.007	0.023

### The Potential Functions of SERPINE1

**Figure 6:**
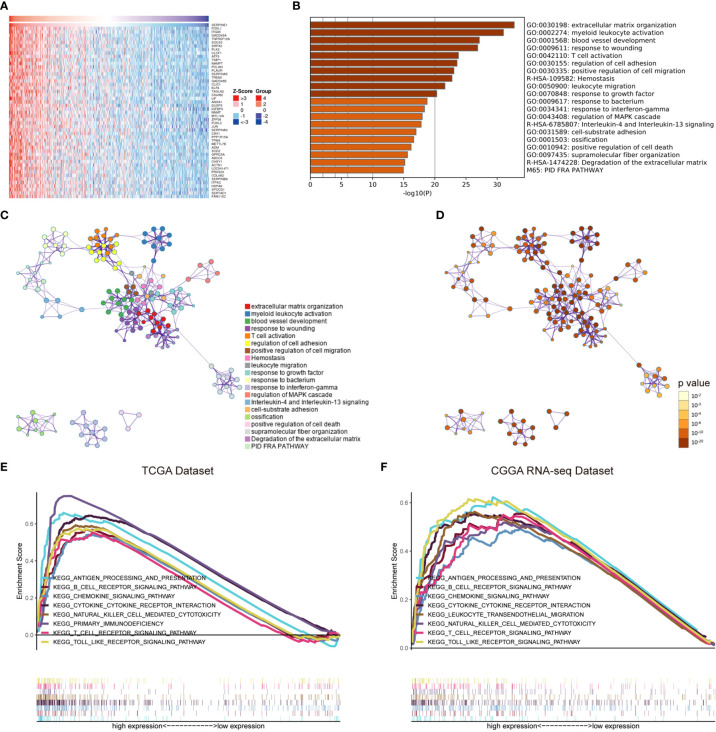
The potential functions of SERPINE1. **(A)** Heatmap of SERPINE1 positively correlated genes. **(B)** Bar graph showing the top 20 enriched terms of SERPINE1 positively correlated genes. The color depth denoted the p-value. Network plot of enriched terms: **(C)** each node represented one enriched term colored by its cluster ID; **(D)** colored by p-value. GSEA enrichment analysis of SERPINE1 in LGG datasets: **(E)** the KEGG pathways enriched in high SERPINE1 expression group of TCGA dataset; **(F)** CGGA dataset. P-value < 0.05 was considered statistically significant.

To better understand the potential functions of SERPINE1, we examined the correlation between SERPINE1 and other genes in the LGG gene expression profile through the online database LinkedOmics (**Figure 6A** and **Supplementary Table 3**) (42). The top 500 positively correlated genes were selected to perform enrichment analysis through the Metascape online tools. As presented in **Figures 6B–D**, these genes were primarily enriched in extracellular matrix organization, myeloid leukocyte activation, blood vessel development, response to wounding, and T cell activation, some of which exhibited immunologic characteristics.

Gene set enrichment analysis (GSEA) was subsequently utilized to distinguish the signaling pathways involved in the LGGs between the high and low SERPINE1 expression groups. Significant difference was demonstrated (FDR<0.05) in the MSigDB collection enrichment (c2.Cp.Keg.v7.2.symbols). As shown in Figures, antigen processing and presentation, B cell receptor signaling pathway, chemokine signaling pathway, cytokine cytokine receptor interaction, natural killer cell mediated cytotoxicity, primary immunodeficiency, T cell receptor signaling pathway, and Toll-like receptor signaling pathway were enriched in the SERPINE1 high expression group from the TCGA cohort (**Figure 6E** and **Supplementary Table 4**). Similar results were obtained in the CGGA dataset (**Figure 6F** and **Supplementary Table 4**), evidently suggesting that SERPINE1 might serve as a crucial factor in regulating immune-related biological processes and pathways in the glioma microenvironment.

### SERPINE1 Regulated the Infiltration of Immune Cells in the LGGs

**Figure 7:**
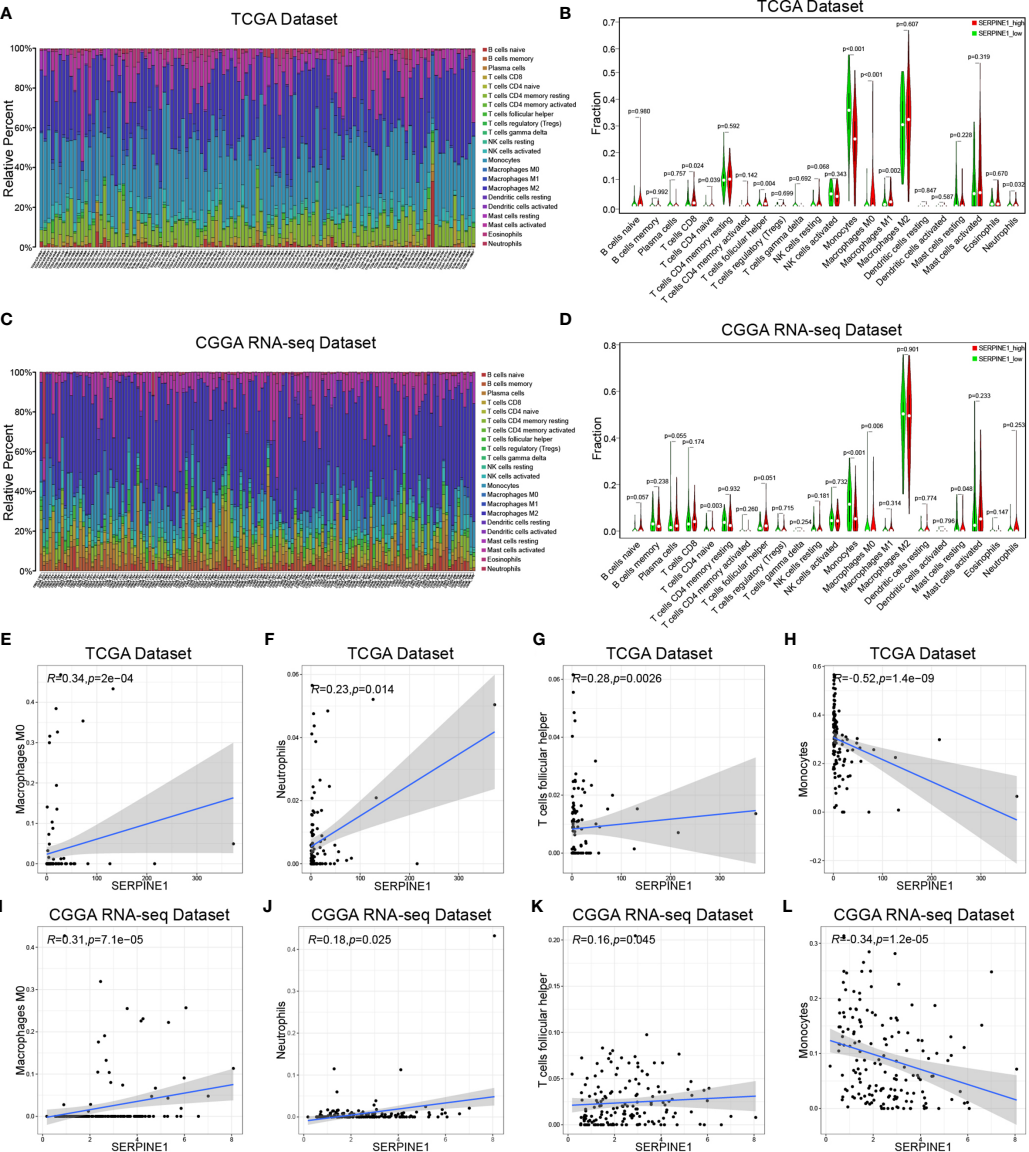
SERPINE1 regulated the infiltration of immune cells in the LGGs. The immune infiltration landscape of LGGs: **(A, C)** bar plots showing the proportion of 22 immunocyte types in TCGA dataset and CGGA dataset. **(B, D)** Violin plots showing the differences in the proportion of 22 immunocyte types between SERPINE1 high and low expression groups. Red colors represented the group with SERPINE1 high expression; green color represented the group with SERPINE1 low expression. The SERPINE1 expression was positively correlated with the infiltration of M0-type macrophages **(E, I)**, neutrophils **(F, J)**, follicular helper T cells **(G, K)**, and negatively associated with monocytes **(H, L)**. P-value < 0.05 was considered statistically significant.

Considering that SERPINE1 might play a role in regulating immune-related responses in the LGGs, the abundance of 22 types of infiltrating immune cells in the TCGA (**Figure 7A**) and CGGA datasets (**Figure 7C**) were examined *via* using the CIBERSORT algorithm. The results showed that NK cells, monocytes, and macrophages accounted for a reasonably large proportion of the 22 immune cell types, which might suggest close involvements of these three cell types in the development of LGGs. And the correlations among 22 types of infiltrating immune cells were weak to moderate in the LGG cohort. Clearly, M2 macrophages presented highly negative correlations with activated mast cells, and eosinophils positively correlated with activated mast cells (**Supplementary Figures 4A, B**). By comparing the proportion of each immune cell type between high- and low-SERPINE1 expression groups, we found that the groups with high-SERPINE1 expression in both of the TCGA and CGGA datasets exhibited relatively low level of monocytes infiltration and high level of infiltration of M0 macrophages and naive CD4+ T cells (**Figures 7B, D**). In addition, Spearman correlation analysis found that SERPINE1 expression was significantly associated with the infiltration of several immune cell types in LGG. In both of TCGA and CGGA datasets, SERPINE1 expression was positively correlated with the infiltration of M0 macrophages (**Figures 7E, I**), neutrophils (**Figures 7F, J**), follicular helper T-cells (**Figures 7G, K**), and negatively correlated with the infiltration of monocytes (**Figures 7H, L**). **Supplementary Figures 4C-J** presented other immune cells related to SERPINE1 expression. Then, we performed ICH staining to identify the content of TAMs (CD68, CD163), neutrophils (CD66b, MPO), monocytes (HLA-DR, CD14), and follicular helper T-cells (CXCR5, ICOS) in LGG tissues. The results showed that the content of TAMs and neutrophils in the PAI-1 high expression group was significantly higher than that in the PAI-1 low expression group (**Supplementary Figures 5A, B**). Between the two groups, there was no significant difference in the infiltration of monocytes and follicular helper T-cells (**Supplementary Figures 5C, D**). Altogether, it was suggested that SERPINE1 expression did influence the immune cell infiltration in the LGG microenvironment, especially the macrophages and neutrophils.

### SERPINE1-Related Inflammatory Responses

**Figure 8:**
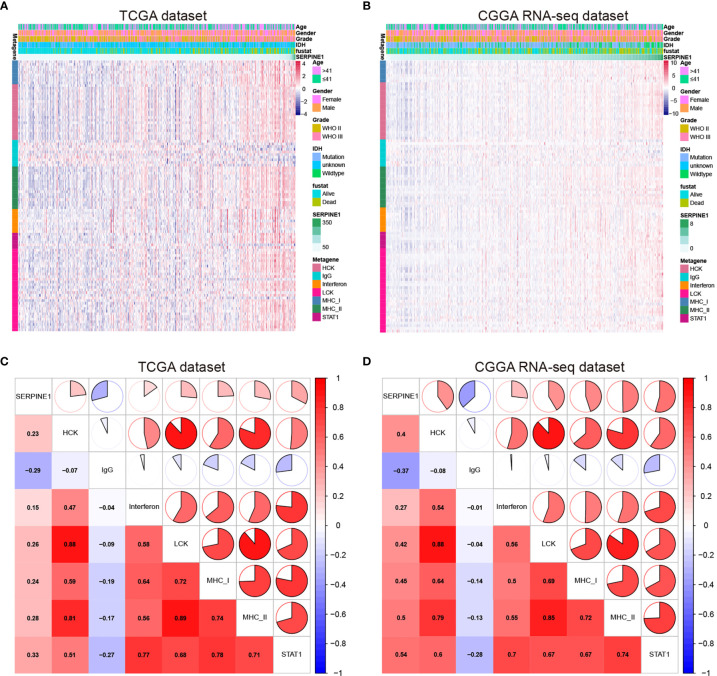
SERPINE1-related inflammatory responses in LGG. **(A, C)** Heatmaps showing the relationship between SERPINE1 and seven metagenes in TCGA and CGGA datasets. **(B, D)** Correlogram showing the correlation between SERPINE1 and seven metagenes in TCGA and CGGA datasets.

Considering the important role of inflammation in host immune reaction to the tumor as well as tumor immunotherapy, we further analyzed the associations between SERPINE1 and different inflammatory responses. Therefore, seven clusters of metagenes (**Supplementary Table 6**), representing different types of inflammatory and immune responses, were selected to analyze the association between SERPINE1 and different inflammatory responses (43). The expression pattern of these metagenes in the TCGA dataset was presented in the **Figure 8A**. As showcased in the heatmap, SERPINE1 expression positively correlates with HCK-, Interferon-, LCK-, MHC_I-, MHC_II-, and STAT1-related genes but negatively with IgG-related genes. To verify the result of heatmap analysis, we convert the expression data of these metagenes into enrichment scores *via* Gene set variation analysis (GSVA). Then, the correlogram was used to display the correlation between seven inflammatory metagene signatures and SERPINE1 (**Figure 8C**). This analysis showed that SERPINE1 was positively related with the signature of HCK, Interferon, LCK, MHC_I, MHC_II, and STAT1 but was negatively associated with IgG, a marker for B lymphocytes activities. Moreover, the analysis based on the CGGA dataset gave identical results (**Figures 8B, D**).

### Correlation Analysis Between SERPINE1 and Immune Checkpoints

**Figure 9:**
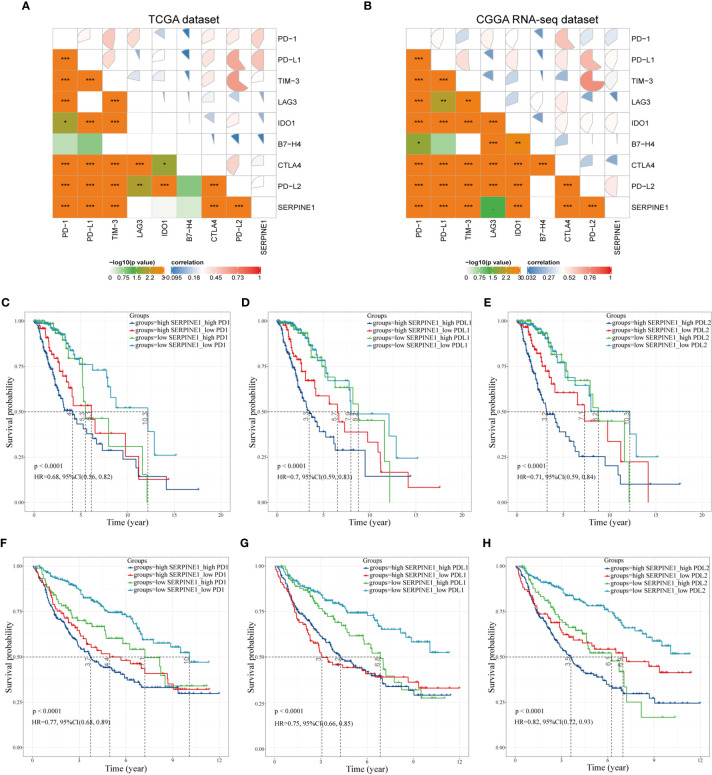
Correlation analysis between SERPINE1 and immune checkpoints. **(A, B)** Correlation matrix plots of SERPINE1 and major immune checkpoints in the TCGA dataset and CGGA dataset. **(C, F)** Kaplan–Meier survival analysis in LGG patients stratified by SERPINE1 and PD-1 expression. **(D, G)** Kaplan–Meier survival analysis in LGG patients stratified by SERPINE1 and PD-L1 expression. **(E, H)** Kaplan–Meier survival analysis in LGG patients stratified by SERPINE1 and PD-L2 expression. P-value < 0.05 was considered statistically significant. *p-value ≤ 0.05; **p-value ≤ 0.01; ***p-value ≤ 0.001.

According to the above study, SERPINE1 was not only identified as an immune-related gene but might affect the infiltration of immune cells in the TME. Considering that suppressive mechanisms in the TME exerted a critical role in the immune evasion of glioma cells (19), we took a step further to explore the potential association between SERPINE1 and some crucial immune checkpoints. Spearman correlation analysis was used to analyze the correlation between SERPINE1 and the immune checkpoint-related genes, including PD-1, PD-L1, PD-L2, CTLA4, TIM-3, IDO1, B7-H4, and LAG3. Correlation matrix plots indicated that SERPINE1 was correlated significantly with several immune checkpoints in the TCGA (**Figure 9A**) and CGGA dataset (**Figure 9B**). Notably, SERPINE1 showed significant positive relationships with PD-1, PD-L1, and PD-L2 both in TCGA and CGGA datasets. Moreover, Kaplan-Meier survival analysis demonstrated that LGG patients with low levels of SERPINE1 and PD1 exhibited appreciably longer overall survival than those with high levels of SERPINE1 and PD1 expression (**Figures 9C, F**). Similar results were obtained in the analysis of SERPINE1 combined with PD-L1(**Figures 9D, G**) and PD-L2(**Figures 9E, H**). In short, these results indicated that SERPINE1 and some immune checkpoints such as PD-1, PD-L1, PD-L2 might act synergistically in the progression of LGGs.

## Discussion

Treatments of LGGs have remained a huge challenge to clinicians due to the aggressive nature and high risk of recurrence (44). Although tremendous efforts have been made to improve the clinical outcome, the prognosis of LGG patients has not been substantially improved in the last decades (45, 46). Therefore, it is necessary to develop novel treatment strategies for glioma patients. In recent years, immunotherapy for gliomas has attracted increasing attention to scientists, based on the continuous in-depth research on the immune TME. For example, some studies have corroborated that combination of PD-1 blockade and local radiotherapy prolonged the survival time of an orthotopic glioma mouse model (47). However, due to the immunosuppressive microenvironment in glioblastoma, PD-1/PD-L1 checkpoint blockades have not made breakthroughs in glioblastoma treatment (48). And the detailed molecular mechanisms of the immune responses in the glioma microenvironment have not been clarified, which greatly limits the development of effective immunotherapies to treat gliomas. This study investigated the TME of LGGs and screened out the prognosis-related immune genes based on the TCGA and CGGA databases, which may provide a new perspective to find potential therapeutic targets for gliomas.

In the screening phase, we used the ESTIMATE algorithm to calculate the immune cell and stromal cell scores for each LGG sample from the TCGA database. We found that high immune or stromal scores tended to predict poor prognosis. WHO grade III or IDH wild-type gliomas, as expected, had higher immune and stromal scores. Similar results were observed in the research of glioblastoma and osteosarcoma (49, 50). These results evidently confirmed that the infiltrative level of immune and stromal cells was correlated with tumor malignancy and prognosis. Subsequently, based on the immune and stromal scores, 1576 differentially expressed genes were screened out. GO and KEGG pathway enrichment analyses further verified a close involvement of these DEGs in the immunologic processes. Additionally, out of the 1576 DEGs, 54 survival-related genes were obtained by the univariate Cox regression analysis, and 46 hub genes were filtered out through the cytoHubba plugin in Cytoscape software. In this way, the target gene SERPINE1 was ultimately selected at the intersection of the survival-related genes and hub genes.

SERPINE1 encodes plasminogen activator inhibitor type 1 (PAI-1), which serves as the primary inhibitor of urokinase plasminogen activator (uPA) and tissue-type plasminogen activator (tPA) (23). Previous studies have primarily focused on its role in thrombosis. However, in recent years, high-throughput sequencing results showed that SERPINE1 was aberrantly overexpressed in various types of tumors. It has also been reported that SERPINE1 was mainly produced by stromal cells in the TME and thus might exert its tumor-promoting function by regulating the interactions between tumor cells and the microenvironment (51). Currently, SERPINE1 as a tumor-promoting factor has been studied in breast cancer, gastric cancer, and head and neck squamous cell carcinoma. For instance, Yang et al. reported that SERPINE1 was an independent predictor of poor prognosis for gastric adenocarcinoma and it promoted tumor cell proliferation, migration, and invasion by regulating epithelial-mesenchymal transition (EMT) (26). Likewise, both *in vivo* and *in vitro* experiments confirmed that SERPINE1 knock-down could inhibit glioma growth and invasiveness (27). Nevertheless, the molecular mechanisms of SERPINE1 were still obscure in gliomas, particularly its regulatory mechanisms in the TME of gliomas.

We subsequently analyzed the expression characteristics of SERPINE1 in the LGGs based on the TGCA, CGGA, and GEPIA2 databases. Compared with the normal brain tissues, SERPINE1 was significantly upregulated in the LGGs. Besides, the expression levels of SERPINE1 were higher in the WHO grade III or IDH wild-type gliomas. According to the previous studies, high grade or IDH wild-type gliomas are often associated with poor prognosis (52). As a result, it was rational to hypothesize that SERPINE1 was a tumor-promoting factor and positively correlated with the malignancy of LGGs. Kaplan-Meier analysis indeed indicated that higher SERPINE1 expression predicted shorter overall survival in the LGG patients. ROC analysis further revealed that SERPINE1 could function as a sensitive indicator predicting one-year, three-year, and five-year survival rates for the LGG patients. Moreover, we found that SERPINE1 was an independent prognostic factor for overall survival using the Cox multivariate analysis. Taken together, we speculated that SERPINE1 was a valuable prognostic biomarker for the LGGs.

To further investigate the potential mechanisms of SERPINE1 in the LGGs, we examined the top 500 genes positively related to SERPINE1 in the LGGs *via* the LinkedOmics website. These related genes were mainly enriched in immune-response related processes, such as myeloid leukocyte activation (GO:0002274), T cell activation (GO:0042110). Besides, gene set enrichment analysis (GSEA) was performed to investigate the biological functions of SERPINE1 in the LGGs based on the TCGA and CGGA database. Likewise, the GSEA results demonstrated that many immune-response related processes existed in the group with high SERPINE1 expression. Taken together, SERPINE1 was an immune-related gene and was involved in the immune processes in the TME of the LGGs. Considering that immune-related genes often conducted their functions by regulating immune cell behaviors, we utilized the CIBERSORT algorithm to assess the proportions of 22 types of immune cells in the microenvironment of LGGs. The results showed that SERPINE1 affected immune cell infiltrations. The group with high expression of SERPINE1 harbored a higher proportion of T cells follicular helper, neutrophils, macrophages M0, and a lower proportion of monocytes. Moreover, we confirmed through ICH staining that TAMs and neutrophils were highly infiltrated in LGG with high PAI-1 expression. As we all know, immune cells are critical components of the TME and have been confirmed to influence tumor behavior and patient prognosis (53). Although tumor-associated microglia/macrophages (TAMs) accounted for the higher proportion of all infiltrating immune cells in the glioma TME, their capacities were not sufficient to cause antitumor immune responses (54). On the other hand, they could secrete copious amounts of anti-inflammatory cytokines to develop an immunosuppressive microenvironment (54, 55). Likewise, neutrophil was known to activate immune response and mediate tissue damage in the inflammatory response, however, tumor-associated neutrophils (TANs) exerted an immunosuppressive role in the TME (56). The activation and recruitment of neutrophils could directly or indirectly affect the recruitment and differentiation of the TAMs, which was important for tumor progression and the maintenance of the TME (57). Moreover, there was initial evidence suggesting that PAI-1 can affect the biological behavior of inflammatory cells. For example, Sakamoto et al. (58) found that PAI-1 that abnormally elevated in esophageal squamous cell carcinoma (ESCC) could promote macrophage infiltration by the Akt and Erk1/2 signaling pathways. And studies indicated that PAI-1 could assist IL-8-mediated neutrophil infiltration *via* inhibiting IL-8/Heparan Sulfate/Syndecan-1 Complex shedding on endothelial cell surfaces (59). In the analysis of SERPINE1 and seven immune metagenes, we found that SERPINE1 expression was particularly correlated with macrophage- and T-cell-related, but not B cell-related immune responses. These results suggested that SERPINE1 is a negative prognostic factor for LGG and plays an important role in the regulation of immune responses. Thus, we speculated that the negative effects of SERPINE1 on the LGGs might be associated with the infiltrations of macrophages and neutrophils.

In this study, SERPINE1, as an immune-related gene, was screened out and was confirmed to affect immune cell infiltrations in the LGG microenvironment. Given the importance of immunotherapy in gliomas, we took a step further to analyze the correlation between SERPINE1 and immune checkpoint genes in the TCGA- and CGGA-LGG datasets. Indeed, SERPINE1 exhibited significant correlations with the immune checkpoints, especially PD-1, PD-L1, and PD-L2, and might synergize with them. It has been demonstrated that the interaction of PD-1 and PD-L1 was a critical mechanism for tumor cells to evade immune surveillance. Blockade of PD-1/PD-L1 could enhance the anti-tumoral T cell immune responses (60). Pembrolizumab and nivolumab, the PD-1 immune checkpoint inhibitors, have received FDA approval for the treatment of metastatic melanoma and non-small-cell lung cancer (61). Moreover, ACT001, which directly targeted PAI-1, has been reported to suppress glioma cell proliferation, migration, and invasion *via* inhibiting the PI3K/AKT pathway (62). Whether ACT001 and pembrolizumab/nivolumab have synergistic effects in the treatment of gliomas will be the subject of our future research.

In summary, we screened SERPINE1 in the immune-related differential genes and further explored its expression features and biological functions in the LGG cohorts through bioinformatic analysis. The results indicated that SERPINE1 could not only act as a prognostic biomarker but also function as a potential therapeutic target for gliomas.

## Data Availability Statement

The datasets presented in this study can be found in online repositories. The names of the repository/repositories and accession number(s) can be found in the article/**Supplementary Material**.

## Author Contributions

XH designed this study and drafted the manuscript. XH, FZ, and DH collected and performed data analysis. XJ, JG, WL, and YW contributed to figures and tables. QL and TX reviewed and edited the manuscript. All authors contributed to the article and approved the submitted version.

## Funding

This work was supported by Natural Science Foundation of China (Grant NO. 81972340, No.81871196, No.81471517), Science and Technology Project of Jinan city (Grant NO. 201907048), Key Projects of Natural Science Foundation of Jiangxi Province (Grant NO. 20192ACB20011), Shandong Provincial Natural Science Foundation, China (Grant No. ZR2018MH005), Shandong Province Key Research and Development Program (Grant No. 2019GSF107046).

## Conflict of Interest

The authors declare that the research was conducted in the absence of any commercial or financial relationships that could be construed as a potential conflict of interest.

## Supplementary Material

Supplementary Figure 1 Relationship between the immune, stromal, ESTIMATE scores and the clinical characteristics of the LGG patients. **(A–C)** The distribution of immune score, stromal score, ESTIMATE score at different ages. **(D–F)** The distribution of immune score, stromal score, ESTIMATE score in different genders. P-value<0.05 was considered statistically significant.

Supplementary Figure 2 Protein-protein interaction (PPI) network of DEGs visualized by Cytoscape software (v3.7.2) (Confidence score=0.97). Node size indicated its connectivity (the larger the node, the more proteins interacted). Red nodes represented upregulated genes, while blue nodes downregulated genes.

Supplementary Figure 3
**(A)** The difference in TIMP1 expression between LGG samples and normal brain samples was not statistically significant. **(B)** SERPINE1 expression was significantly higher in LGG samples than in normal brain samples. (*represent p-value<0.05) **(C)** The batch effect between mRNAseq_693 dataset and mRNAseq_325 dataset from the CGGA database was removed by the “sva” R package. **(D, F)** The expression level of SERPINE1 mRNA in LGG at different ages. **(E, G)** The expression level of SERPINE1 mRNA in LGG in different genders. P-value<0.05 was considered statistically significant.

Supplementary Figure 4
**(A, B)** Correlation matrix plots of 22 immunocyte types in TCGA dataset and CGGA dataset. **(C–E)** Immune cells related to SERPINE1 expression in TCGA dataset. **(F–J)** Immune cells related to SERPINE1 expression in CGGA dataset. P-value<0.05 was considered statistically significant.

Supplementary Figure 5
**(A)** The biomarkers of TAMs (CD68, CD163) were detected by immunohistochemistry staining in LGG samples. **(B)** The biomarkers of neutrophils (CD66b, MPO) were detected by immunohistochemistry staining in LGG samples. **(C)** The biomarkers of monocytes (CD14, HLA-DR) were detected by immunohistochemistry staining in LGG samples. **(D)** The biomarkers of follicular helper T-cells (CXCR5, ICOS) were detected by immunohistochemistry staining in LGG samples.
