# Supplementary figures and images for: Immune-Related Gene SERPINE1 Is a Novel Biomarker for Diffuse Lower-Grade Gliomas via Large-Scale Analysis
Source: Front Oncol. 2021 May 20;11:646060. doi: 10.3389/fonc.2021.646060 (PMC8173178; doi:10.3389/fonc.2021.646060)

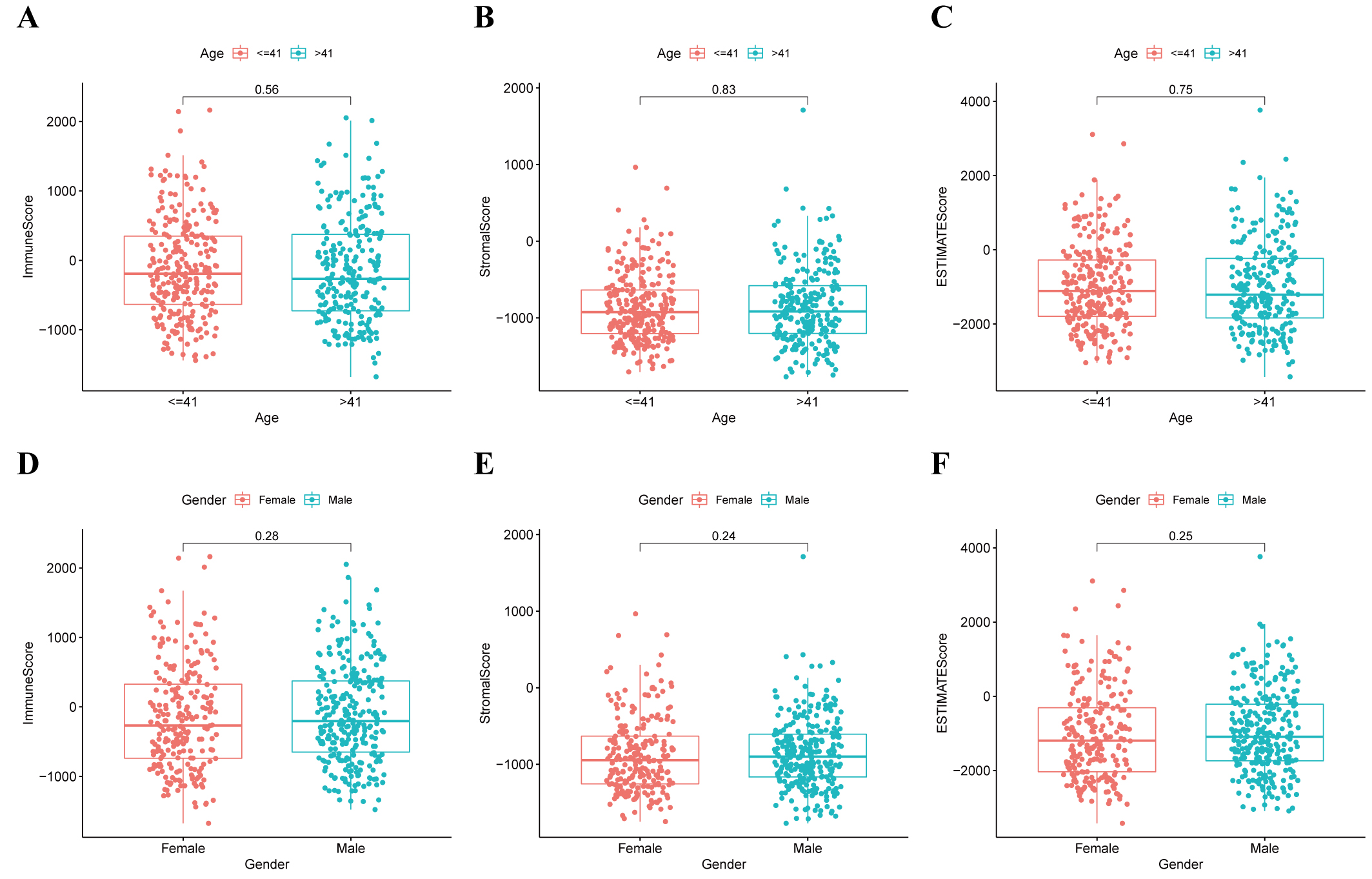

Supplement: Supplementary Figure 1 — Relationship between the immune, stromal, ESTIMATE scores and the clinical characteristics of the LGG patients. (A–C) The distribution of immune score, stromal score, ESTIMATE score at different ages. (D–F) The distribution of immune score, stromal score, ESTIMATE score in different genders. P-value<0.05 was considered statistically significant. [file Image_1.jpeg]

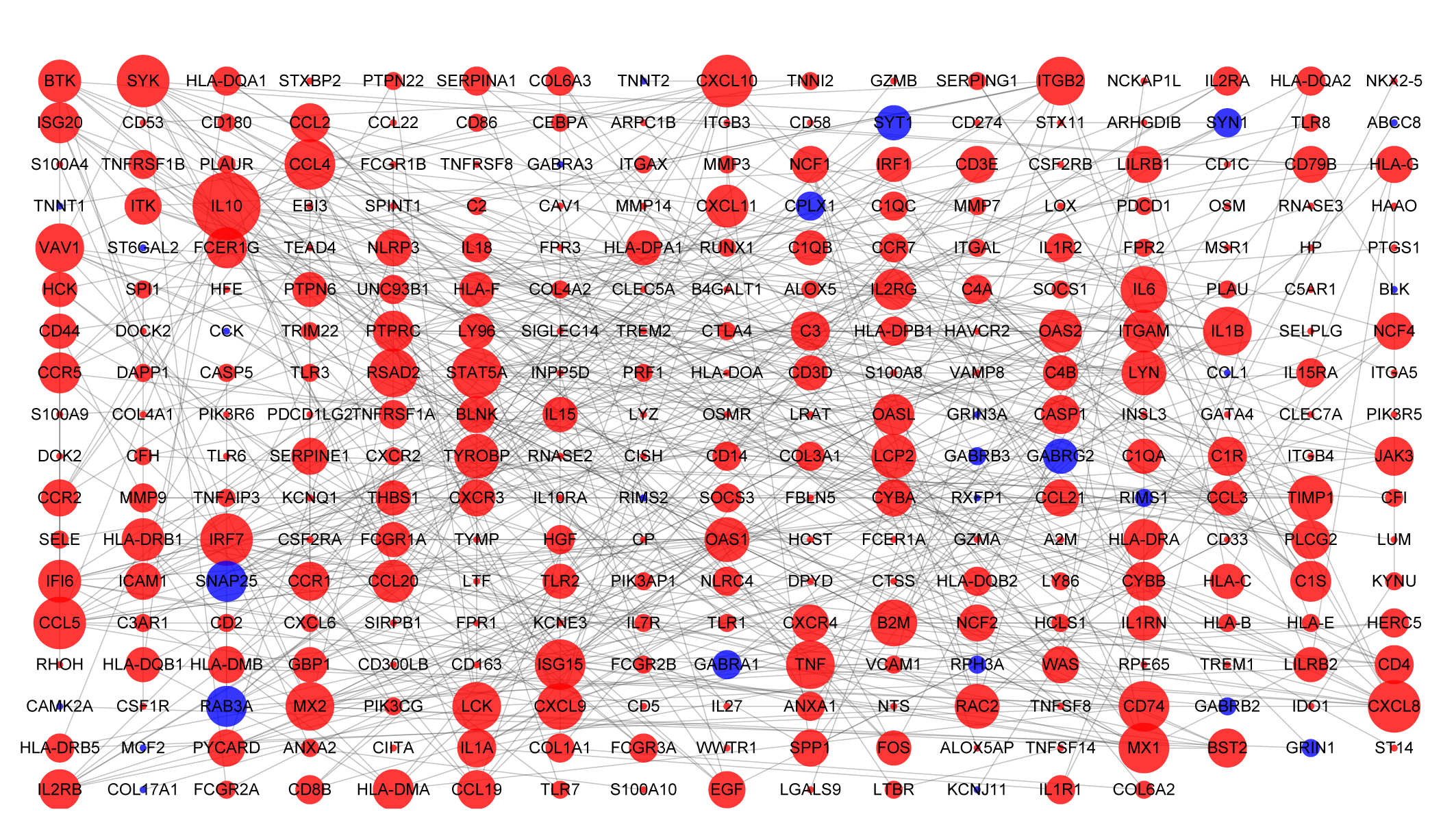

Supplement: Supplementary Figure 2 — Protein-protein interaction (PPI) network of DEGs visualized by Cytoscape software (v3.7.2) (Confidence score=0.97). Node size indicated its connectivity (the larger the node, the more proteins interacted). Red nodes represented upregulated genes, while blue nodes downregulated genes. [file Image_2.jpeg]

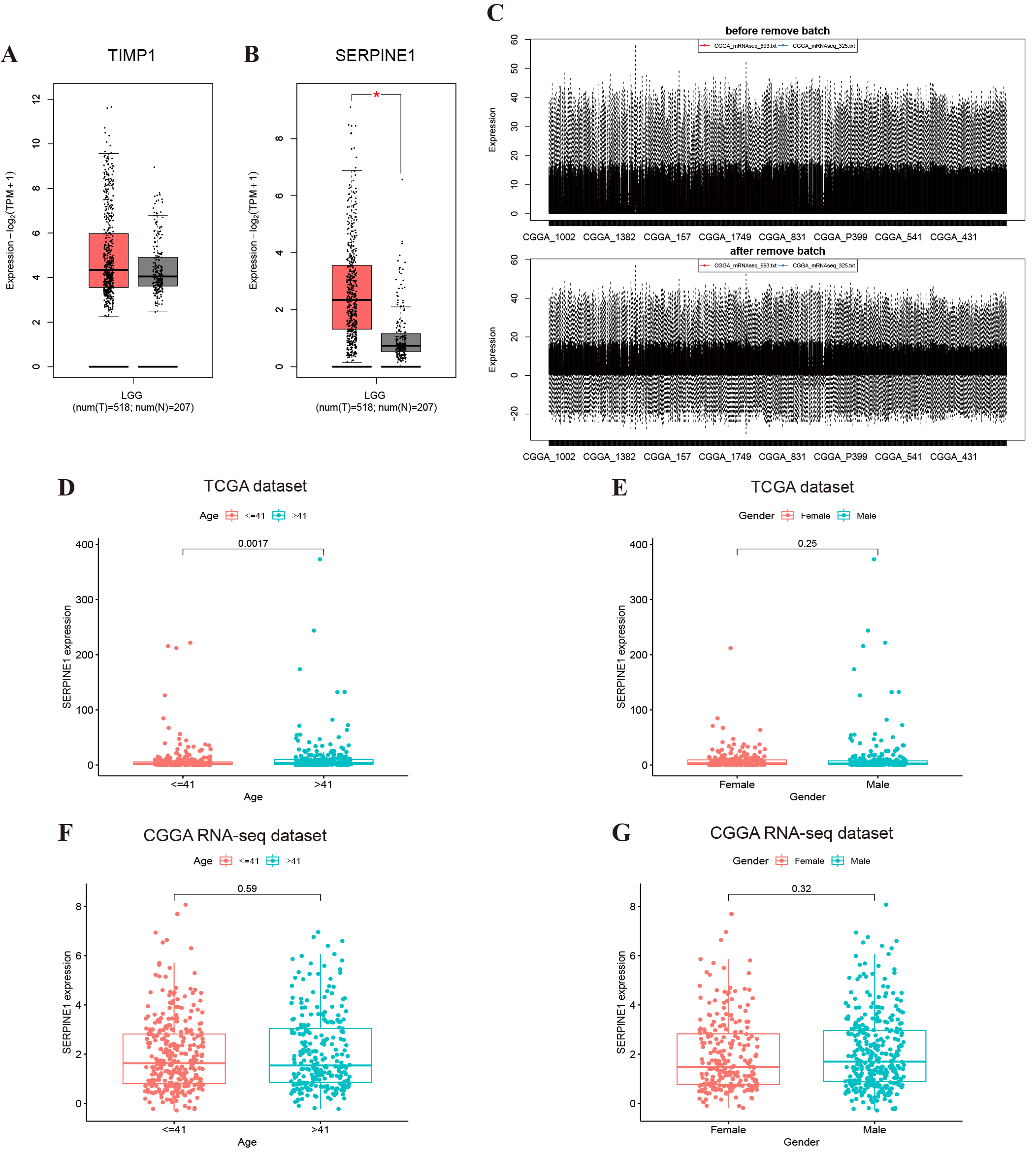

Supplement: Supplementary Figure 3 — (A) The difference in TIMP1 expression between LGG samples and normal brain samples was not statistically significant. (B) SERPINE1 expression was significantly higher in LGG samples than in normal brain samples. (*represent p-value<0.05) (C) The batch effect between mRNAseq_693 dataset and mRNAseq_325 dataset from the CGGA database was removed by the “sva” R package. (D, F) The expression level of SERPINE1 mRNA in LGG at different ages. (E, G) The expression level of SERPINE1 mRNA in LGG in different genders. P-value<0.05 was considered statistically significant. [file Image_3.jpeg]

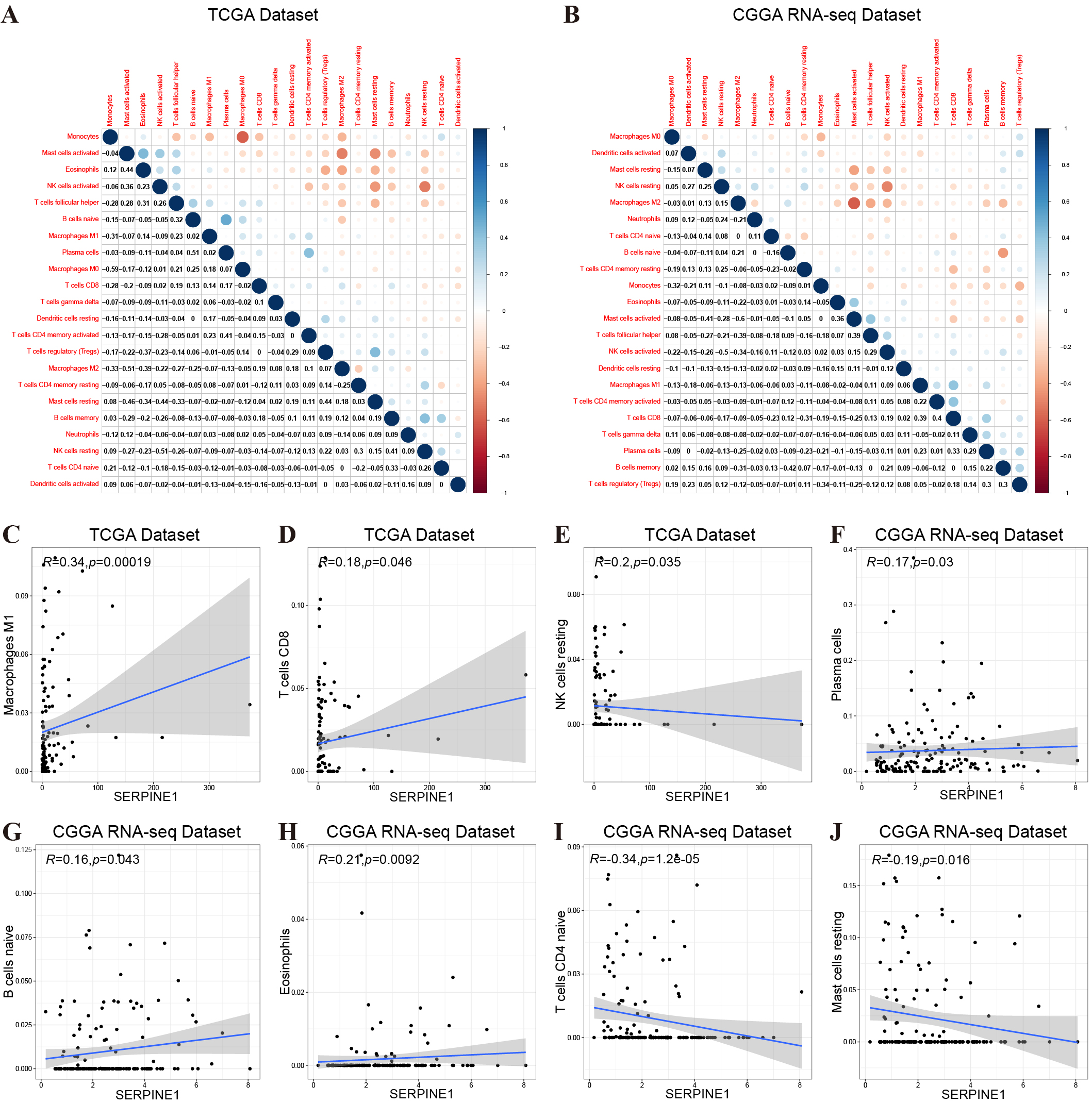

Supplement: Supplementary Figure 4 — (A, B) Correlation matrix plots of 22 immunocyte types in TCGA dataset and CGGA dataset. (C–E) Immune cells related to SERPINE1 expression in TCGA dataset. (F–J) Immune cells related to SERPINE1 expression in CGGA dataset. P-value<0.05 was considered statistically significant. [file Image_4.jpeg]

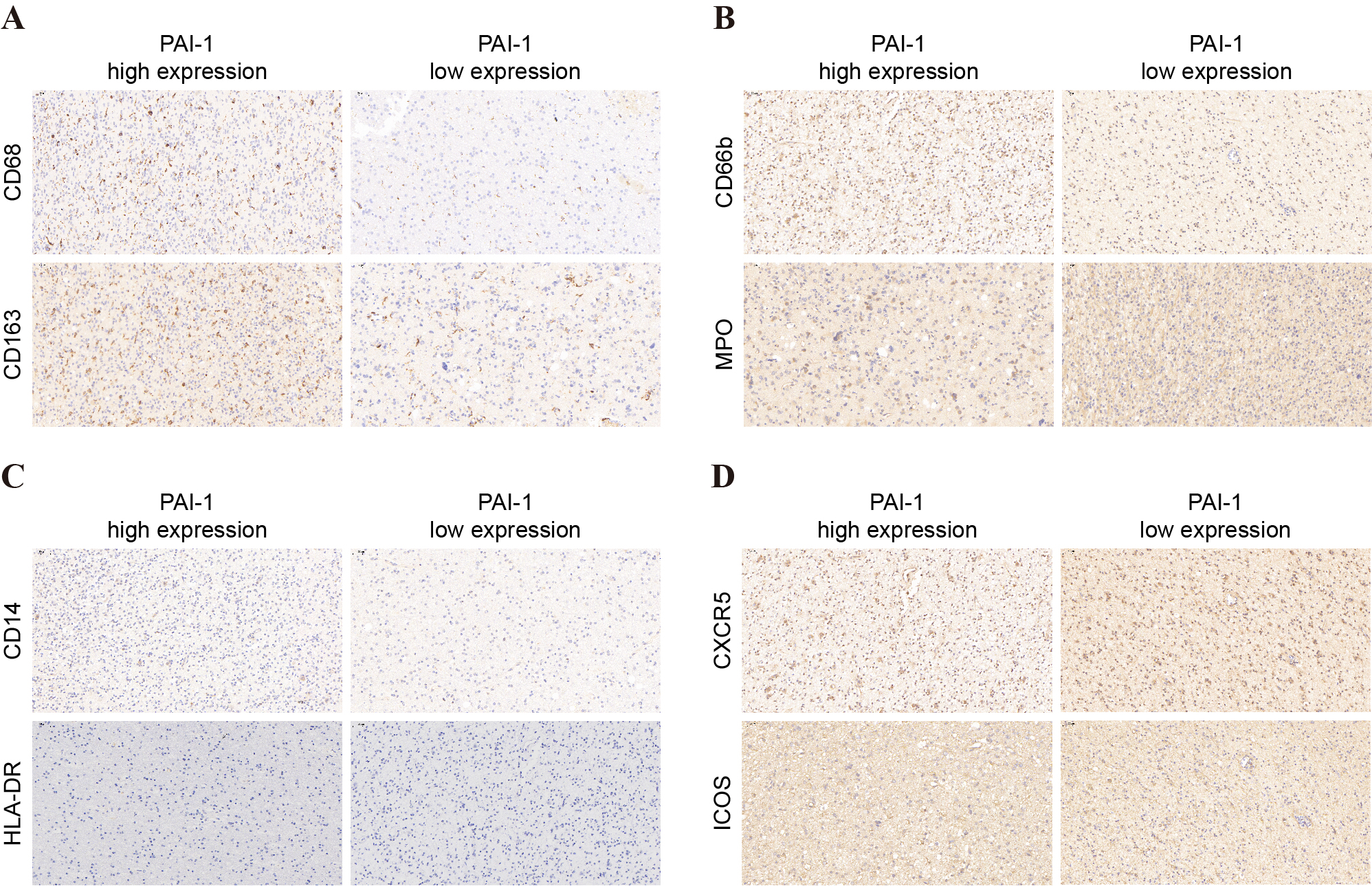

Supplement: Supplementary Figure 5 — (A) The biomarkers of TAMs (CD68, CD163) were detected by immunohistochemistry staining in LGG samples. (B) The biomarkers of neutrophils (CD66b, MPO) were detected by immunohistochemistry staining in LGG samples. (C) The biomarkers of monocytes (CD14, HLA-DR) were detected by immunohistochemistry staining in LGG samples. (D) The biomarkers of follicular helper T-cells (CXCR5, ICOS) were detected by immunohistochemistry staining in LGG samples. [file Image_5.jpeg]
