# Supplementary material for: Immune-Related Gene SERPINE1 Is a Novel Biomarker for Diffuse Lower-Grade Gliomas via Large-Scale Analysis
Source: Front Oncol. 2021 May 20;11:646060. doi: 10.3389/fonc.2021.646060 (PMC8173178; doi:10.3389/fonc.2021.646060)
Supplement: Supplementary file 7 [file Table_4.docx]

Table 4 Gene sets enriched in the high SERPINE1 expression phenotype

| Datasets | Gene set name | NES | p-value | FDR |
| --- | --- | --- | --- | --- |
| TCGA | KEGG_ANTIGEN_PROCESSING_AND_PRESENTATION | 1.96 | 0.002 | 0.004 |
|  | KEGG_B_CELL_RECEPTOR_SIGNALING_PATHWAY | 1.93 | 0.000 | 0.005 |
|  | KEGG_CHEMOKINE_SIGNALING_PATHWAY | 1.92 | 0.004 | 0.005 |
|  | KEGG_CYTOKINE_CYTOKINE_RECEPTOR_INTERACTION | 2.14 | 0.000 | 0.001 |
|  | KEGG_NATURAL_KILLER_CELL_MEDIATED_CYTOTOXICITY | 2.09 | 0.002 | 0.001 |
|  | KEGG_PRIMARY_IMMUNODEFICIENCY | 1.95 | 0.004 | 0.004 |
|  | KEGG_T_CELL_RECEPTOR_SIGNALING_PATHWAY | 1.91 | 0.002 | 0.005 |
|  | KEGG_TOLL_LIKE_RECEPTOR_SIGNALING_PATHWAY | 1.98 | 0.000 | 0.003 |
|  |  |  |  |  |
| CGGA | ANTIGEN_PROCESSING_AND_PRESENTATION | 1.82 | 0.008 | 0.028 |
|  | KEGG_B_CELL_RECEPTOR_SIGNALING_PATHWAY | 1.77 | 0.004 | 0.037 |
|  | KEGG_CHEMOKINE_SIGNALING_PATHWAY | 1.75 | 0.006 | 0.038 |
|  | KEGG_CYTOKINE_CYTOKINE_RECEPTOR_INTERACTION | 1.80 | 0.012 | 0.030 |
|  | KEGG_LEUKOCYTE_TRANSENDOTHELIAL_MIGRATION | 1.87 | 0.002 | 0.028 |
|  | KEGG_NATURAL_KILLER_CELL_MEDIATED_CYTOTOXICITY | 1.75 | 0.008 | 0.039 |
|  | KEGG_T_CELL_RECEPTOR_SIGNALING_PATHWAY | 1.82 | 0.000 | 0.030 |
|  | KEGG_TOLL_LIKE_RECEPTOR_SIGNALING_PATHWAY | 1.91 | 0.004 | 0.024 |

NES: normalized enrichment score; FDR: false discovery rate
